# Non-parametric tests in detecting glaucoma progression

**DOI:** 10.22336/rjo.2017.39

**Published:** 2017

**Authors:** Anca Pantalon, Crenguța Feraru

**Affiliations:** * Ophthalmology Clinic, “Sf. Spiridon” University Hospital, Iași, Romania; ** Surgery Department, Ophthalmology Unit, “Gr. T. Popa” University of Medicine and Pharmacy, Iași, Romania

**Keywords:** glaucoma, progression, non-parametric analysis

## Abstract

Automated perimetry still represents the gold standard in long term glaucoma monitoring. On a daily practice basis, glaucoma progression analysis could be difficult due to the long time needed to detect, confirm, and quantify the progression rate. Moreover, “trend” and “event” analysis require a good theoretical basis to perform and interpret.

**Aim of study** was to present an alternative method to conventional Glaucoma Progression Analysis (Humphrey Visual Field Analyzer, Carl Zeiss® Inc.) applied for the early detection of glaucoma progression. Such an “event” analysis orients the clinician in a fast manner on the progression profile in glaucoma patients and might adapt the follow up visits accordingly.

**Method and material:** 41 eyes from 41 patients with open angle glaucoma were studied in a longitudinal manner, over a 24 months’ time interval from diagnosis.

**Results:** in the GPA analysis, a positive “event” (progression) was detected in 11/ 41 eyes (26.82%). Non-parametric analysis confirmed progression in all GPA cases, and additionally found 8 more eyes with positive progression (46.34% studied eyes). Mc Nemar concordance analysis between tests was good and relevant (kappa index k=0.596, p=0.000), with positive correlation (r=0.652, p=0.008).

In **conclusion**, NPA tends to overestimate the number of progression cases in a cohort, but it can easily orient the clinician on the profile of the followed patients. In the first years, the GPA analysis can be highly inaccurate, but there is a great need to detect which patients are at significant risk for vision loss (fast progressors). Yet, combining the two methods of detection of glaucoma progression, the practitioners might direct their selected interest and attention towards observing a larger than expected number of patients who are at risk for vision loss over time due to glaucoma, but not necessarily in a fast manner.

## Introduction

Standard automated perimetry (SAP) remains the “gold standard” method to assess functional glaucomatous damage and progressive disease. Recent improvements in the Swedish interactive thresholding algorithm (SITA) strategy and the guided progression analysis (GPA) have further settled it as the preferred method for diagnosis and follow-up of glaucomatous functional loss [**1**].

On a daily practice basis, glaucoma progression analysis could be difficult due to the long time needed to detect, confirm, and quantify the progression rate. Moreover, “trend” and “event” analysis require a good theoretical basis to “integrate into the clinical context” and interpret the information. As such, clinical decisions are based on more aspects than a visual field output. In this respect, more and more research is directed towards adjunctive methods of visual function assessment and progression detection (such as the non-parametric tests) in eyes with visual field loss. These types of alternative analysis could earlier direct the attention towards a selected category of patients with higher progression risk and could also enable a faster profiling for progression in some cases or “modulate” clinical decisions for the doctors in terms of good clinical practice. 

“Event” analysis detects progression and it is developed from all the re-test values measured in the follow up visual field (VF) examinations, in locations with a given baseline value. Because patients included in a clinical trial are usually highly variable in response, significant deviation from the baseline is often met. Applying the non-parametric tests helps ensuring that progression is not flagged because of deviant values in highly variable individuals, improving the overall specificity [**2**]. 

The aim of the study was to present an alternative method to conventional Glaucoma Progression Analysis (Humphrey Visual Field Analyzer, Carl Zeiss® Inc.) applied for the early detection of glaucoma progression. Such an “event” algorithm (NPA) applied to the mean deviation (MD) orients the clinician in a fast manner on the progression profile in glaucoma patients and might adapt the follow up visits accordingly.

Study design: longitudinal prospective study on 41 eyes from 41 patients with primary open angle glaucoma. Subjects were enrolled over a period of 2 years (2012-2014), and then followed for the next 2 years (2014-2016) in our Glaucoma Unit at “Sf. Spiridon” University Hospital, Iasi, Romania.

Ethics: our study was performed in respect with the Declaration of Helsinki. The Ethical Review Board of “Gr. T. Popa” University of Medicine and Pharmacy approved the study and each patient signed an informed consent. 

## Material and method

We included only patients newly diagnosed with primary open angle glaucoma (POAG), according to EGS criteria European Glaucoma Society [**3**]. POAG was defined in the presence of open anterior chamber angle on gonioscopy, glaucomatous optic disc damage on clinical examination (focal or diffuse neuroretinal rim thinning, localized notching, or nerve fiber layer defect) and corresponding visual field (VF) defects. Glaucoma severity was graded according to Hodapp criteria [**4**].

In Standard Automated Perimetry (24-2 SITA Standard SAP, Humphrey Field Analyzer II, Carl Zeiss Meditec Inc., Dublin, CA, USA), VF changes for glaucoma were defined if at least two of the three Anderson’s criteria were fulfilled (three or more non-edged points in a cluster depressed to P<5% and one of which depressed to P<1%, Glaucoma Hemifield Test outside normal limits and pattern standard deviation depressed to P<5%). Reliability of tests was assessed. Tests with fixation losses, false-positive or false-negative rates >20% were considered unreliable and excluded from the analysis. A minimum number of 5 valid VF tests were required for each patient in our study.

All reliable VF tests were analyzed for progression by Glaucoma Progression Analysis (GPA) software, which provides both an event-based and a trend-based progression analysis. Both analyses took the first two reliable VF tests as baseline landmark. For GPA, visual field progression was based on glaucoma change probability maps [**5**]. In glaucoma change probability maps, the threshold value of each test point location in every follow-up field is compared with a mean of the values from the same test point in 2 baseline fields. Points that have changed more than might be expected from random variability at P<0.05 are flagged as significantly changing. To limit the effect of increasing media opacities, GPA uses pattern deviation probability plots [**6**]. Likely progression is reached when 3 or more test point locations at any location in the field, not necessarily contiguous, show a significant deterioration in 3 consecutive tests. Possible progression occurs when 3 or more such locations have been identified in 2 consecutive tests [**7**,**8**]. Nonparametric progression analysis (NPA) is based on an algorithm ranking MD values during the follow up interval (see algorithm in the table below). 

**Table 1:** Criteria for “suspected”, “possible” and “likely” progression (adapted after WGA Consensus, 2011)

	“Suspected” progression	“Possible” progression	“Likely” progression
GPA	>/ = 3 empty triangles (white)*	>/ = 3 half empty triangles	>/ = 3 full triangles (black)
NPA	1 VF for follow up with MD lower than lowest MD of baseline fields	2 consecutive VF for follow up with MD lower than lowest MD of baseline fields	3 consecutive VF for follow up with MD lower than lowest MD of baseline fields
*>/ = 3 open triangles are quite common by chance and thus hardly indicative for progression; therefore “possible progression” requires confirmation in a shorter interval with GPA [**2**]			

During the follow up, if one MD value appeared to be better than baseline in NPA analysis, the analysis up to that point was declared null (“stationary” patient) and a new baseline needed to be settled. If 5 valid VF did not remain after this “new baseline” settlement, patient was excluded from the study.

NPA can be performed directly from the printouts, without additional software. Classifications by GPA and by NPA were compared at the end of the follow-up period.

Quantitative assessment of VF decay over time was made by linear regression (Trend) analysis of the mean deviation (MD) changes over time; slopes of progression (decibels/ year) based on threshold maps and its level of significance (p-values) were calculated. 

Patients were followed at every 4 months, when identical tests were performed. We excluded non-compliant patients or those with significant lens opacities, ocular comorbidities, refractive errors > 5D spherical and > 3D cylinder.

If both eyes were eligible, only one was chosen based on the worse MD level at baseline. At baseline, clinical parameters were collected from the charts and included in our study: age, gender, best corrected visual acuity (BCVA) by ETDRS chart, intraocular pressure (IOP) by Goldmann tonometer, central corneal thickness (CCT) by ultrasonic pachymeter (DGH-550, DGH Technology Inc., Exton, PA, USA), C/ D ratio (Volk 78D lens), number of topical medications, VF test parameters. VA, IOP, and VF tests were repeated at each follow up visit.

The majority of the patients required topical therapy, but no surgical intervention (laser or incisional procedure-trabeculectomy) was performed during the follow up period. During monitoring, treatment was modified if the IOP was not efficiently controlled; IOP level was individually set, according to glaucoma severity, risk factors and life span. 

**Statistical analysis**

A SPSS 18.0 statistical software (SPSS Inc. Chicago, IL, USA) was used to process the data. Descriptive statistics analyzed demographics, follow up time, MD, PSD and IOP values. The MD slopes and corresponding residuals (a measure of inter-test variability within a subject) were calculated by using linear regression analysis. The proportion of eyes that “likely” progressed by GPA or NPA was compared. Agreement between analyses was assessed (McNemar test, agreement coefficient “κ”). Continuous variables were compared by using t test and proportions using χ2 statistic. Statistical significance was defined at the p <0.05 level. Sensitivity is the ability of a test to correctly classify an individual as “diseased”, likely progressors in our case, whereas the ability of a test to correctly classify an individual as *disease- free* is called the test’s specificity. Sensitivity and specificity were calculated according to standard statistical definitions [**9**]. Also we calculated the NPA capacity to detect real progression by using positive predictive value (PPV), that can tell if the results obtained by NPA analysis could be as reliable as the GPA analysis (“gold standard”).

## Results

The mean age in our group was 64.46 +/ -8.5 years, with a clear female sex predominance 85.37% females (37 eyes) to 14.63% males (6 eyes). Calculated spherical equivalent was slightly hyperopic +0.70+/ -1.55D. A better description of the studied population can be followed in the table below (**Table 2**). 

**Table 2:** Baseline parameters in the study

Parameters (baseline)	POAG
VA (decimal)	0.82+/ -0.21
IOP baseline (mmHg)	16.69+/ -4.58
No. medications	1.68+/ -1.19
CCT (µm)	537.73+/ -27.20
**C/ D ratio** (clinical assessment)	0.66+/ -0.17
OCT examination	
C/ D ratio (vertical)	0.7+/ -0.13
Disk area (mm2)	2.02+/ -0.38
Neural rim area (mm2)	0.93+/ -0.26
RNFL thickness (µm)	78.88+/ -12.94
CGL thickness (µm)	74.44+/ -10.20
VF examination	
MD (db)	-2.37+/ -3.24
PSD (db)	2.56+/ -1.83

Later evolution of parameters and comparison between baseline and final values can be followed in **Table 3**.

**Table 3:** Baseline vs. final parameters in the study

Parameter	Initial	Final	p (test t)
VA (decimal)	0.82+/ -0.21	0.73+/ -0.24	0.000
IOP (mmHg)	16.69+/ -4.58	14.04+/ -3.70	0.000
No. medications	1.68+/ -1.19	2.26+/ -1.28	0.001
MD (dB)	-2.37+/ -3.24	-3.96+/ -4.04	0.013
PSD (dB)	2.56+/ -1.83	3.65+/ -2.63	0.007

**Fig. 1:**
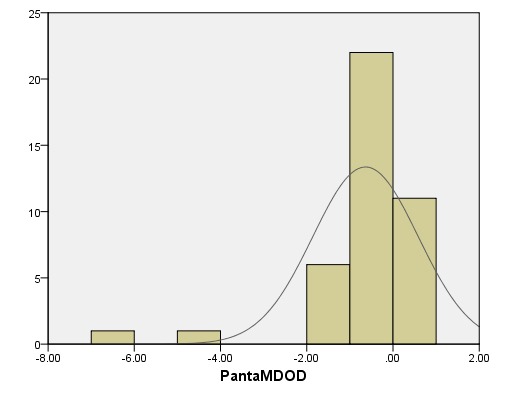
Histogram of MD values in the studied POAG patients

**Fig. 2:**
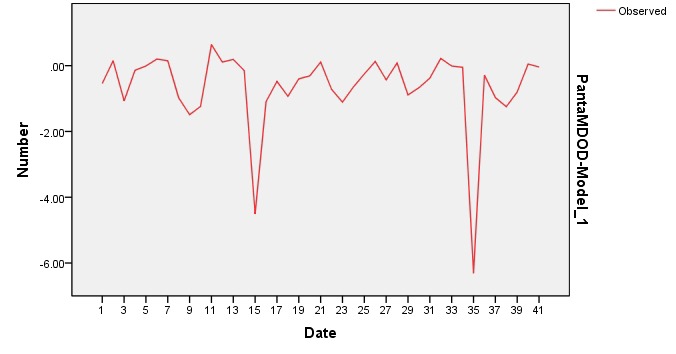
MD slopes in all 41 eyes in the study

The overall visual field decay calculated after 24 months of follow up was -0.63 +/ - 1.12 dB/ year. The histogram of all MD values for the 41 eyes included in the study is represented in **Fig. 1**. MD slopes are depicted in **Fig. 2** for all followed eyes.

**Fig. 3:**
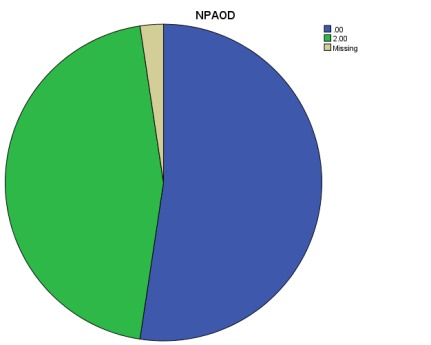
“Likely” progression flagged by NPA analysis

**Fig. 4:**
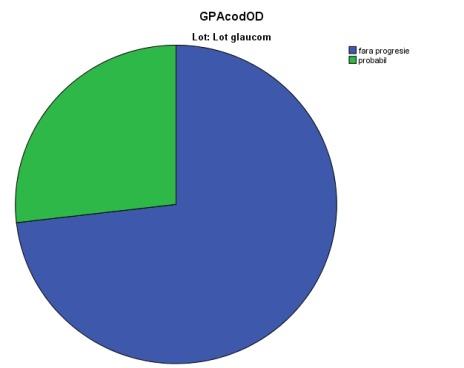
“Likely” progression flagged by GPA analysis

A simple calculation of VF decline does not also reflect the degree of clinical and statistical significance. Therefore, we evaluated the presence of “likely” glaucoma progression (“event” analysis), through two different methods, independently and saw the agreement between them. For this, we followed the algorithm described in the “Material and Method” section above (**Table 1**). Applying the above-mentioned criteria, we found in our study that there was a considerable higher proportion of likely progressive cases by NPA analysis than GPA analysis (see **Fig. 3**-**4**).

**Fig. 5:**
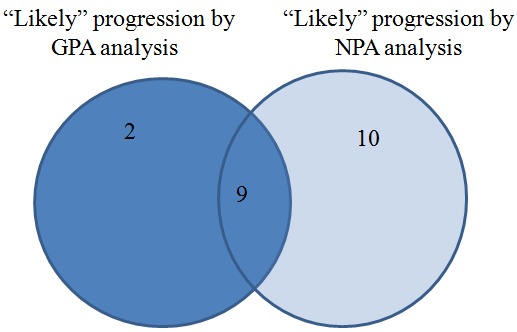
“Likely” progression analyzed by NPA and GPA methods in the study

11 eyes were flagged as “likely progressive” by GPA analysis (26.8% eyes), whereas 19 eyes were found as “likely progressive” glaucoma damage by NPA analysis (46.34% eyes); percentages were statistically different, p<0.01. 9 eyes with a “likely” progression by GPA analysis were flagged similarly by NPA analysis, whereas 2 cases were not confirmed by NPA. A better understanding of the “likely progression” cases may be followed in the Venn diagrams below (**Fig. 5**).

The mean MD slope for the 11 progressive cases by GPA was -1.74+/ -1.87 dB/ year, compared to -1.83+/ -1.96 dB/ year with a positive progression by NPA analysis. The difference was not statistically significant, p>0.05.

Kappa agreement index between the two methods (GPA/ NPA) was k=0.596, p=0.000 (Mc Nemar test), with positive medium-strong correlation (Pearson test): r=0.652, p=0.008.

Since for detecting progression SAP remains the “gold standard”, we calculated the sensitivity and specificity of NPA in relation to this. For the present study, we found that in the first two years of follow up, GPA sensitivity was 84.61% and specificity was 66.66% in our glaucoma patients. In a similar manner, we calculated the NPA sensitivity= 65.09% and NPA specificity= 90%. Positive predictive value (PPV) for GPA was 52.38%, compared to 90.47% NPA.

## Discussions

In this study, we prospectively observed 41 eyes of 41 patients with POAG who were followed up by using SAP. We compared a nonparametric ranking method applied to the MD (NPA) with GPA. Most eyes flagged as showing likely progression by GPA were detected by NPA as well. In addition, some eyes were flagged as showing progression by NPA but not by GPA. Because of its design, GPA is insensitive to a general decrease in sensitivity. The development of cataract is presumably a common cause of a general decrease in sensitivity. Insensitivity to cataract is obviously an advantage in a glaucoma trial. However, in a clinical setting, patients may benefit from the fact that a clinician has to evaluate the lens before he or she can interpret perimetry results. Yet, glaucoma deficit might include diffuse loss in addition to localized deterioration, case in which GPA will not detect the changes. As such, NPA detects what GPA cannot. Some eyes were flagged by GPA without being flagged by NPA. This is not an unexpected finding because agreement between different progression detection algorithms has been shown to be less than perfect [**12**-**16**]. The agreement in this study is in line with previously published values (k=0.37 for the EMGT vs. subjective assessment [**13**] and k=0.40 for the Advanced Glaucoma Intervention Study vs. glaucoma change probability [**15**].

Other drawbacks of GPA are that additional software is needed, all visual fields involved must be stored in a single perimeter (which is often challenging), and it is only available for more recent versions of the Humphrey field analyzer (Carl Zeiss Meditec Inc). For these reasons, a subjective evaluation of a series of fields is still the most widely used approach today. However, inter-observer agreement for this approach is moderate at best [**10**,**11**]. Therefore, a new approach that is simple to use and understand, objective, not dependent on software, applicable for all (static-automated) perimeters, and applicable at all disease stages, was introduced herein. This method applies nonparametric ranking to the global index mean deviation (MD) - “nonparametric progression analysis” (NPA). 

The mean rate of progression in our study was -0.63 +/ - 1.12 db/ year, which is in good agreement with the rate of progression in other studies [**17**-**20**]. This rate of progression was reached with a mean intraocular pressure of 15 mm Hg during our follow-up period, similar to the intraocular pressure values in the treated arm of the EMGT study. Neither GPA nor NPA provides information regarding the speed of deterioration. Hence, after progression was diagnosed by using either technique, the amount of deterioration and its localization (toward fixation or not) should be evaluated. The algorithms warn the clinician that something is going wrong rather than telling the whole story. 

In clinical practice, we advice combining the two methods discussed above in order to increase both sensitivity and specificity. In the current study, the “gold standard” in perimetric progression – GPA is more sensitive in detecting an event similar to progression than specific enough to eliminate the “false alarms”. A false positive is essentially a false alarm, which could lead to subjects being incorrectly referred, causing them unnecessary anxiety and wasting clinical time and resources, or worse still, in other pathologies, potentially having to undergo unnecessary treatment if the correct diagnosis is not made in the clinic. A false negative can have equally dire consequences; if a patient has the pathology but is not diagnosed, then this would lead to him being falsely reassured that all is well and not receiving appropriate treatment at the earliest stage of his disease [**21**].

In this respect, for the current study, an easy manual method – NPA might help improve this aspect and increase the specificity in detection glaucoma progression.

## Conclusions

NPA tends to overestimate the progressive number in a cohort, but its purpose is to alert and orient the clinician towards the possibility of progression in the followed population. As shown in this study, NPA is an easy tool for screening likely progression in glaucoma; it can be used with any perimeter at any disease stage, without the need for additional software. NPA cannot substitute the GPA methods, but GPA analysis can be highly inaccurate, especially in the first two years due to marked learning effects and high variability in responses during tests. As such, authors suggest combining the two methods for a better glaucoma management. Since nonparametric progression analysis seems to flag more eyes as showing progression than GPA, clinicians should carefully consider their decisions before any decisions and obtain confirmations through the same method and also additional ones. 
